# Genomics driven-oncology: challenges and perspectives

**DOI:** 10.1186/s12885-015-1147-7

**Published:** 2015-03-23

**Authors:** Nicola Normanno, Ian A Cree

**Affiliations:** 1 Cell Biology and Biotherapy Unit, Istituto Nazionale Tumori “Fondazione Giovanni Pascale”, IRCCS, Napoli, Italy; 2 Department of Pathology, University Hospital Coventry and Warwickshire, Coventry, CV2 2DX, UK

**Keywords:** Cancer genomics, Lung cancer, RET, Targeted therapy

## Abstract

Molecularly defined subgroups of tumors characterized by specific driver mutations have been identified in the majority of cancers. The availability of novel drugs capable of targeting signaling pathways activated by genetic derangements has led to hypothesize the possibility to treat patients based on their genomic profile. A clear example is represented by lung adenocarcinoma for which it has been possible to identify driver genetic alterations in approximately 75% of the cases. Among these, RET fusion transcripts are detectable in about 1–2% of lung adenocarcinomas and might represent targets for therapeutic intervention with RET kinase inhibitors. However, a number of issues need to be addressed to make genomics-driven oncology routinely accessible for cancer patients, including: 1) the availability of novel methods in molecular diagnostics that allow a comprehensive molecular characterization of lung tumors starting from a low input DNA/RNA; 2) identification of reliable and reproducible biomarkers of response/resistance to targeted agents; 3) the assessment of the role of tumor heterogeneity in the response to drugs targeting molecular pathways.

## Background

Somatic alterations of genes involved in the regulation of cell proliferation, differentiation and survival play a pivotal role in the pathogenesis and progression of the majority of human cancers. In many cancer types it has been possible to identify molecularly defined subgroups of tumors that are characterized by driver mutations (genetic alterations causally associated with carcinogenesis) that are often mutually exclusive [1]. An example is represented by the adenocarcinoma subgroup of non-small-cell lung cancer (NSCLC), in which using high throughput technology it has been possible to identify driver genetic alterations in approximately 75% of the cases [2].

The identification of such driver mutations and the availability of novel drugs capable of targeting signaling pathways activated by genetic derangements has led to hypothesize the possibility to treat patients based on their genomic profile (Table 1). An example of this potential approach, defined genomics driven-oncology, is represented by RET rearrangements in lung cancer [3]. Chimeric RET proteins generated by chromosomal rearrangements leading to RET fusion transcripts have been identified in ~1–2% of lung adenocarcinomas but might represent as many as 6–19% of tumors from never-smokers without other driver mutations [3]. Response to treatment with RET inhibitors such as vandetanib or cabozantinib has been reported in selected cases [4-7]. Phase 2 clinical trials of RET kinase inhibitors in lung cancer patients harboring RET rearrangement are ongoing (Table 2). In this regard, a retrospective analysis of RET translocations, gene copy number gains and expression from four randomized trials of vandetanib in NSCLC is published in this issue of BMC Cancer [8]. Noteworthy, this is the first series of patients from clinical trials that have been extensively screened for RET molecular alterations, although retrospectively.

**Table 1:** **Selected genetic alterations representing potential biomarkers in lung adenocarcinoma and related drugs in clinical development**

Biomarker	Drug
EGFR mutations*	Gefitinib/Erlotinib/Afatinib
ALK rearrangements*	Crizotinib
ROS-1 rearrangements	Crizotinib
RET rearrangements	Cabozantinib/Vandetanib/Ponatinib
NTRK1 rearrangements	Cabozantinib
MET amplification	Crizotinib/Cabozantinib
NRAS mutations	Selumetinib/Trametinib
ErbB-2 mutations/amplification	Lapatinib/Trastuzuma/Afatinib
KRAS mutations	Selumetinib/Trametinib
BRAF V600E	Vemurafenib/Dabrafenib
BRAF Y472C	Dasatinib

*approved.

**Table 2:** **Phase II clinical trials of RET tyrosine kinase inhibitors in RET**-**rearranged lung carcinoma**

Study identifier	Drug(dose)	Molecular targets	Primary outcome measure
NCT01639508	cabozantinib (60 mg/day)	MET, VEGFR2, FLT3, c-KIT, AXL and RET	ORR*
NCT01823068	vandetanib (300 mg/day)	VEGFR2, EGFR, RET and FGFR-1	ORR
NCT01813734	ponatinib (45 mg/day)	ABL, FLT3, KIT, FGFR, PDGFR, VEGFR2 and RET	ORR

*objective response rate.

## Discussion

While the absolute number of RET-positive tumors reported in the paper by Platt et al. [8] is too low to draw any firm conclusion, this study provides important insights of critical discussion that can be generalized to the entire field of genomics-driven oncology:

1. The rate of RET rearrangement in NSCLC was as low as 0.7%, while previous studies have reported frequencies up to 6%. RET rearrangements have been suggested to be more frequent in Asian patients, in non-smoker and in the adenocarcinoma subgroup. Therefore, the selection of the study population might significantly affect the frequency at which RET rearrangements are detected. In addition, RET rearrangement is usually mutually exclusive to other driver mutations, and its frequency results higher in patients that do not harbor the more frequent KRAS and EGFR mutations [3]. Nevertheless, this observation poses a major problem for molecular diagnostics that is common to many cancer types. In fact, the number of potential predictive biomarkers that might offer possibility of therapeutic intervention in lung cancer as well as in other tumor types is increasing exponentially (Table 1). Identification of driver mutations might result in a survival advantage for cancer patients that have access to novel drugs through clinical trials or, in selected cases, to receive an off-label treatment with agents approved for other indications [9]. However, the time, the cost, and the amount of tissue needed for a wide molecular profiling using routine diagnostic methods are not compatible with the standard clinical workup, in particular in lung cancer. In many European countries diagnosis of lung cancer is based in over 50% of the cases on cytology samples or small biopsies that might not be sufficient for analysis of somatic mutations and gene rearrangements in several different genes using sequencing, Real Time PCR and/or FISH. Indeed, a 26.9% failure rate in FISH analysis due to an inadequate number of tumor cells or sample quality was reported by Platt and colleagues [8]. This observation underlines the need for novel methods in molecular diagnostics that allow a comprehensive molecular characterization of lung tumors in the routine clinical workout [10]. In this respect, different methods to detect mutations and fusions using genotyping or targeted next generation sequencing are being explored, and might be ready in a short timeframe for clinical implementation [11-14].
2. Oncogenic pathways can be activated by different molecular mechanisms. Indeed, Platt and colleagues found RET amplification in 2.8% of the cases, low RET gene copy number gain in 8.1%, and RET protein expression in 8.3% [8]. Although the relative low number of positive cases prevents any firm conclusion, the finding that the response rate to vandetanib did not correlate with any of these markers does suggest that they have little predictive utility to identify those patients who will benefit from vandetanib therapy. This observation has two important implications. First, activation of RET through amplification or low copy number gain might not represent a driver molecular alteration in lung cancer. In addition, based on the data presented in this issue of BMC Cancer by Platt et al. [8], immunohistochemical detection of RET protein is not a surrogate of RET rearrangement. In fact, RET protein expression was found in cases that did not harbor RET rearrangement. Surprisingly, no RET protein expression was found in some cases with RET rearrangement. This might be due to technical limitations of the immunohistochemistry protocol. Nevertheless, it might be worth to explore whether protein expression levels might affect response to RET inhibitors in patients with RET rearranged lung tumors.
3. Only three RET-rearrangement positive patients received vandetanib treatment in the trials reported by Platt et al. [8], and this low number does not allow to make any conclusion regarding association of vandetanib treatment with efficacy in the RET rearrangement positive subpopulation. However, it is quite surprising that none of the three vandetanib-treated RET-rearrangement-positive patients had an objective response. Importantly, one patient received a 100 mg dose of vandetanib in the ZODIAC trial. Two patients were treated with the standard 300 mg dose of vandetanib in the ZEPHYR study, and both showed radiologic evidence of tumor shrinkage although a response could not be confirmed at the next visit. The ongoing phase 2 clinical trial of vandetanib in lung cancer patients harboring RET rearrangement is employing a 300 mg dose and will clear out the efficacy of the drug in this subgroup of patients (Table 2). Nevertheless, we have come to expect that a tumor harboring a driver mutation will respond to a specific inhibitor, which was not the case in this study. On the other hand, increasing evidence suggests that tumors, including lung carcinoma, are heterogeneous: many tumors contain several clones of neoplastic cells that accumulate during tumor progression different molecular alterations, which might represent mechanisms of resistance to target-based agents [15]. Therefore, it is possible that even tumors with a driver mutation show resistance to specific inhibitors. Indeed, the response rate of lung cancer patients with EGFR mutations to EGFR tyrosine kinase inhibitors ranged between 56% and 86% in different clinical trials, thus suggesting that primary resistance is a phenomenon common to different tumors with driver mutations [16]. In this regard, a more comprehensive molecular characterization of lung tumors might allow to better identify those patients that will benefit from specific targeted agents.

## Conclusion

Identification of relatively rare mutations is transforming tumors with high incidence such as lung adenocarcinoma in some rare diseases, each characterized by a specific molecular alteration. Molecular classification of lung tumors based on driver mutations represents a major challenge for molecular diagnostics, but also an important opportunity for cancer patients to access to novel drugs. In this regard, a number of issues need still to be addressed to make genomics-driven oncology routinely accessible for cancer patients.
